# Enteric Fever and Milk Supply

**Published:** 1875-10

**Authors:** 


### Enteric Fever and Milk Supply.

Dr. E. Duncan had prepared a careful report on the recent epidemic of enteric fever in Crosshill and Eaglesham. He commences by explaining, in terms which can be generally understood and appreciated by the non-professional portion of the public, how this disease may be spread; one of his main objects being to remove the general ignorance prevailing on this subject, in the hope that, by so doing, the public may exhibit more interest in sanitary questions. Dr. Duncan at the onset expresses the conviction that enteric fever does not arise *de novo,* and that the cases in which the disease does appear to originate simply from the inhalation of decomposing animal matter, are really instances in which the poison has been communicated by some article of food; a method of infection illustrated by his report. The outbreak in question commenced in the month of January last, in Crosshill, near Glasgow. It appeared to die out after the middle of February, but recurred with renewed violence after that date, about 280 cases occurring up to the end of March, in a population not exceeding 14,000. The inquiry into the means by which the disease was spread appears to have been conducted by a process of exclusion, the various conditions favoring such spread being considered separately. The nature of the soil is first dealt with, reference being made to Prof. von Pettenkofer's views on this subject; but consideration of this subject led the writer to conclude that the soil had no connection either with the origin or the spread of the fever. The prevailing atmospheric conditions are next briefly adverted to, and also set aside. The various conditions of sewerage and drainage are dwelt with at some length; and although defects were found, as might have been expected, yet, in many of the houses where the disease appeared, some of those defects which are so much associated with the spread of this disease were absent, and in other houses, which were not affected, grave defects were discovered. In short, it is evident from Dr. Duncan's description, that the epidemic was not caused by defects of sewerage, although it was probably to some extent propagated by them. With regard to the water supply, it does not appear to have been at fault; and it is specially pointed out by Dr. Duncan that although the water supply is an intermittent one, yet, since the water closets are in every case supplied by means of a cistern, such pollution of water as arose at Caius College and at Lewes cannot have taken place. The pollution to which water may be subjected in the mains is, however, by no means limited to suction of foul matters from closet-pans in the manner in which it occurred at the two places named. Foul and even excrementitious matters can be drawn into the mains from the soil which they traverse, wherever there is a cracked or faulty pipe, and the supply is an intermitting one; the filth being taken up by the water, or deposited in the house cistern; and we have but little doubt that some of the deposit in our London cisterns reaches the mains in this way. It does not, however, appear that any such mishap caused the epidemic under discussion, there being an absence of any evidence implicating the water supply. Finally, Dr. Duncan considers the question of the milk supply. This subject is examined at considerable lenght, and the result of the investigation tends in the strongest manner to show that the disease was spread through the agency of this article of diet. Enteric fever has prevailed for some time in a certain district whence a considerable portion of the milk supply was derived, and it had attacked the families of farmers whose milk was conveyed to the affected locality. Indeed, out of a total of 262 families receiving milk from what we may now term from the suspected dairies, 94 were attacked with enteric fever; whereas, out of 242 families receiving other milk supplies, only 18 were affected, and of these 18 families it was ascertained, on subsequent inquiry, that 10 got occasional supplies from the suspected dairies. With regard to the remainder of these exceptional cases, it is only a matter of surprise that the number was so small, in view of the large area over which the epidemic was spread.

With regard to the method in which the milk of the various farmers became infected, we cannot now follow Dr. Duncan throughout the many details with which he deals; but one example is worth recording. In November last, enteric fever attacked the family of a dairyman at Eaglesham. On his premises was a dung-stead which partly drained towards and into two wells, the contents of which were used for the dairy and farm purposes, and which were also drunk by persons residing in a back row. In December, the residents of this row were attacked with enteric fever, the stools of as many as twenty patients being thrown into adjoining privies. Constant soakage must have taken place from the privies towards and into a streamlet close by; but just at this date the privy contents were frozen up and the soakage was thus stayed for five weeks, at the end of which time a thaw ensued, rain came on and the accumulated contents of the now specifically diseased privies soaked and drained towards the stream. This water course, which also receives some of the drainage from the dung-stead above refered to, formed the principle source of water for another dairy farm; and, following on this thaw, both the farmer's family and the persons residing within the area of the distribution of his milk became affected with enteric fever. This indicates very clearly the wisdom and propriety of a sanitary survey of farms from which a town milk supply is drawn; such, for instance, as is regularly instituted by the Aylesbury Dairy Company for all its farms.

The whole history of this epidemic has been carefully worked out by Dr. Duncan, and a perusal of his report will repay those who are interested in such investigations. His views concerning the spread of this outbreak are also confirmed by the opinion of Dr. Littlejohn, who promises a further report on the subject, but who has already, as the result of an inquiry made on behalf of the Board of Supervision, expressed his conviction that the importation of the disease into the affected district had been conclusively traced to the use of an infected milk supply.—*British Med. Jour.*, *July* 10, 1875.—*Monthly Abstract.*

